# *EZH2* dysregulation: Potential biomarkers predicting prognosis and guiding treatment choice in acute myeloid leukaemia

**DOI:** 10.1111/jcmm.14855

**Published:** 2019-12-03

**Authors:** Ming‐qiang Chu, Ting‐juan Zhang, Zi‐jun Xu, Yu Gu, Ji‐chun Ma, Wei Zhang, Xiang‐mei Wen, Jiang Lin, Jun Qian, Jing‐dong Zhou

**Affiliations:** ^1^ Department of Respiratory Medicine Affiliated People’s Hospital of Jiangsu University Zhenjiang Jiangsu China; ^2^ Department of Hematology Affiliated People’s Hospital of Jiangsu University Zhenjiang Jiangsu China; ^3^ Zhenjiang Clinical Research Center of Hematology Zhenjiang Jiangsu China; ^4^ The Key Lab of Precision Diagnosis and Treatment in Hematologic Malignancies of Zhenjiang City Zhenjiang Jiangsu China; ^5^ Laboratory Center Affiliated People’s Hospital of Jiangsu University Zhenjiang Jiangsu China

**Keywords:** AML, expression, *EZH1*, *EZH2*, mutation

## Abstract

Accumulating studies have proved *EZH2* dysregulation mediated by mutation and expression in diverse human cancers including AML. However, the expression pattern of *EZH2* remains controversial in acute myeloid leukaemia (AML). *EZH1/2* expression and mutation were analysed in 200 patients with AML. *EZH2* expression was significantly decreased in AML patients compared with normal controls but not for *EZH1* expression. *EZH2* mutation was identified three of the 200 AML patients (1.5%, 3/200), whereas none of the patients harboured *EZH1* mutation (0%, 0/200). *EZH2* expression and mutation were significantly associated with −7/del(7) karyotypes. Moreover, lower *EZH2* expression was associated with older age, higher white blood cells, *NPM1* mutation, *CEBPA* wild‐type and *WT1* wild‐type. Patients with *EZH2* mutation showed shorter overall survival (OS) and leukaemia‐free survival (LFS) than patients without *EHZ2* mutation after receiving autologous or allogeneic haematopoietic stem cell transplantation (HSCT). However, *EZH2* expression has no effect on OS and LFS of AML patients. Notably, in *EZH2* low group, patients undergone HSCT had significantly better OS and LFS compared with patients only received chemotherapy, whereas no significant difference was found in OS and LFS between chemotherapy and HSCT patients in *EZH2* high group. Collectively, *EZH2* dysregulation caused by mutation and under‐expression identifies specific subtypes of AML *EZH2* dysregulation may be acted as potential biomarkers predicting prognosis and guiding the treatment choice between transplantation and chemotherapy.

## 1 INTRODUCTION

Acute myeloid leukaemia (AML) represents a heterogeneous myeloid malignancy with considerable variability especially in cytogenetically and molecular signatures as well as clinical outcome.^1^, ^2^ Despite the tremendous progress has been made in the treatment of AML, clinical outcome of these patients still remains unsatisfactory.^1^, ^2^ Because AML is a highly heterogeneous disease, its treatment needs to be more personalized and precise based on the risk classifications.^2^, ^3^ Until now, cytogenetic abnormalities and molecular alterations provide the most powerful prognostic information.^2^ Karyotypes of t(15;17), t(8;21), t(16;16) and normal karyotype with double *CEBPA* mutation or isolated *NPM1* mutation identified at the diagnosis time of AML usually predict favourable outcome, whereas −5/5q−, −7/7q−, t(6;9), inv(3), t(9;22), t(v;11q23), complex and *FLT3* mutation indicate poor outcome.^2^ These patients with high risks surely need intensive therapy especially haematopoietic stem cell transplantation (HSCT) to improve survival.^2^ Consequently, identification of novel biomarkers which could predict outcome or guide treatment choice will make more contribution to the clinical management of AML.

Epigenetic dysregulation is hallmark of blood cancers especially in AML.^4^, ^5^ Located on the chromosome 7q36.1, *EZH2* gene (Enhancer of Zeste homologue 2) encodes a key member of the *PRC2* (polycomb repressive complex 2) and mediates transcriptional inactivation through di‐ and trimethylation of lysine 27 of histone H3 (H3K27me2/3).^6^ Accumulating studies have proved the phenomenon of *EZH2* dysregulation in diverse human cancers.^7^ Evidence showed that *EZH2* may have a dual role in cancer development, acting as a tumour suppressor or an oncogene depending on the type of cancer.^8^ Overexpression of *EZH2* was observed in numerous solid tumours, and targeting *EZH2* can cause regression of carcinogenesis.^9^, ^10^, ^11^ However, *EZH2* inactivation medicated by mutation or under‐expression in myelodysplastic syndromes (MDS) or myeloproliferative neoplasms (MPN) can contribute to disease pathogenesis and is associated with a poor prognosis.^12^, ^13^, ^14^, ^15^ In AML, *EZH2* mutation was associated with −7/del(7q) and low bone marrow blast percentage but not affected prognosis.^16^ Recently, Zhu et al showed that overexpression of *EZH2* was a frequent event and was associated with extramedullary infiltration in AML.^17^ In addition, *EZH2* silencing resulted in decreased proliferation and migration ability and increased apoptosis, suggesting its oncogenic role in AML.^17^ However, Göllner et al demonstrated that loss of the histone methyltransferase *EZH2* induced resistance to multiple drugs in AML, indicating it may play as tumour suppressor gene in AML.^18^ These contradictory results have aroused our concern and interest to further explore *EZH1/2* expression and mutation in patients with AML.

## 2 MATERIALS AND METHODS

### 2.1 Patients

In this study, we analysed the 200 adult AML patients (173 patients with RNA‐seq data, 194 patients with methylation data and 200 patients with mutation data) from TCGA (The Cancer Genome Atlas) database.^19^ All AML patients were received induction chemotherapy, consolidation treatment included chemotherapy (100 patients) and HSCT (73 patients) as reported.^19^ In addition, to compare the difference of these patients with normal controls, GEPIA (http://gepia.cancer-pku.cn/detail.php) were also used.^20^ The study protocol was approved by the Washington University Human Studies Committee, and informed consents were obtained from all patients.

### 2.2 Bioinformatics analyses

The details were reported as our previous study.^21^

### 2.3 Statistical analyses

SPSS 22.0 and GraphPad Prism 5 were used for statistical analyses and figures creation. Mann‐Whitney's *U* test and Pearson chi‐square analysis/Fisher's exact test was applied for the comparison of continuous variables and categorical variables. The prognostic effect of *EZH2* mutation/expression on leukaemia‐free survival (LFS) and overall survival (OS) analysed through Kaplan‐Meier analysis and Cox regression analysis. The two‐tailed *P* value <.05 in all statistical analyses was defined as statistically significant.

## 3 RESULTS

### 3.1 EZH1/2 expression and mutations in AML

**Figure 1:**
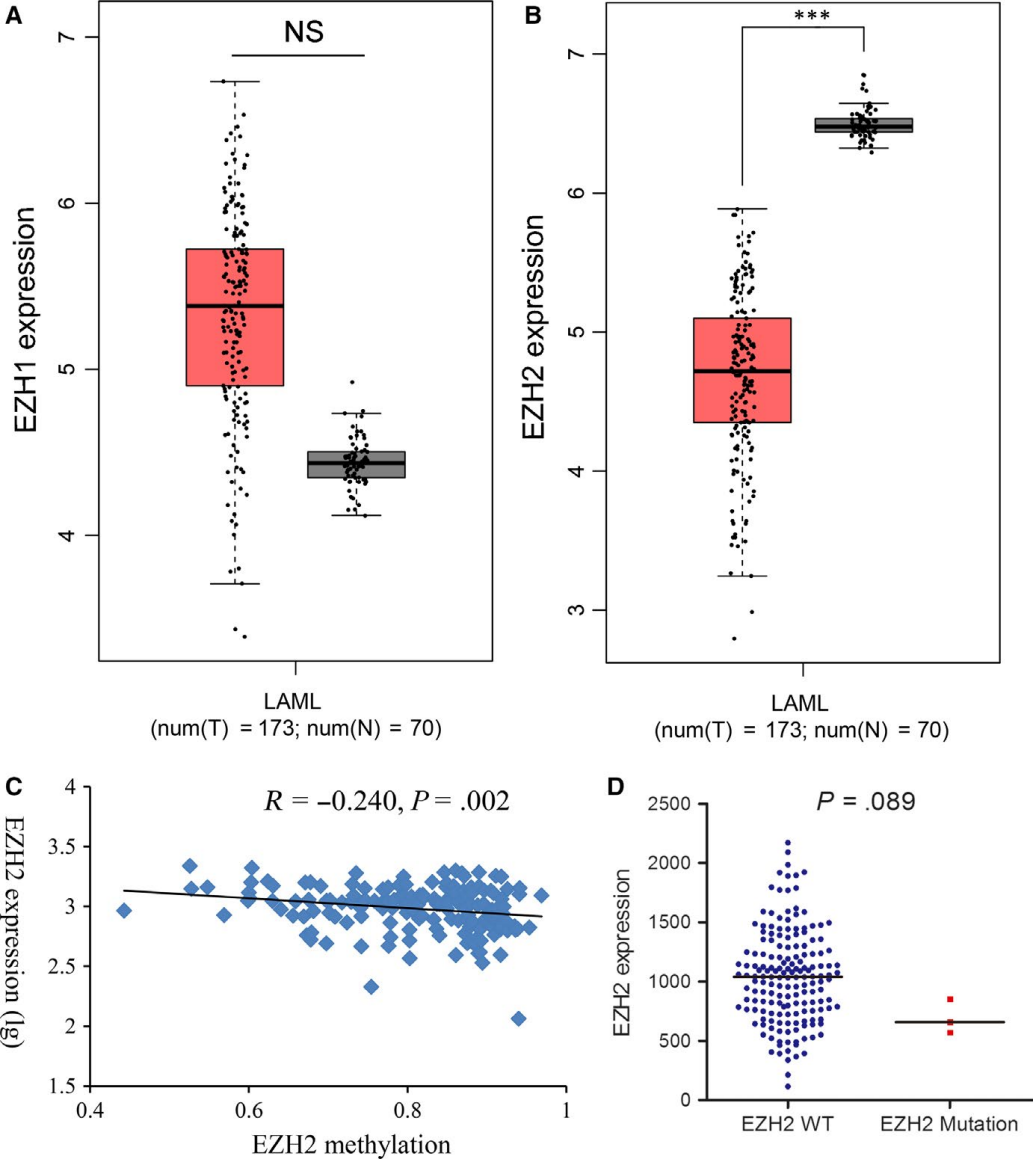
*EZH1/2* expression in AML. A, B, *EZH1/2* expression in normal controls and AML patients from TCGA datasets analysed through the GEPIA (http://gepia.cancer-pku.cn/detail.php). C, Correlations of *EZH2* expression with *EZH2* methylation in AML. D, *EZH2* expression in AML patients with and without EZH2 mutation. P‐values were calculated using the Mann‐Whitney *U* test. The correlation was analysed by Spearman correlation test. NS: no significance; ***: *P* < .001

A cohort of 200 AML patients from public TCGA datasets was used for differential expression analysis. *EZH1/2* expression was available in 173 patients. Using the GEPIA (http://gepia.cancer-pku.cn/detail.php), we found *EZH2* expression was significantly decreased in AML patients compared with normal controls (*P* < .001, Figure 1B), but *EZH1* expression showed no difference (*P* > .05, Figure 1A). No association was observed between *EZH1* and *EZH2* expression in AML patients (*R* = −.020, *P* = .791). In addition, *EZH1/2* methylation was available in 194 patients. No association was found between *EZH1* methylation and expression in AML patients (*R* = −.118, *P* = .126). However, *EZH2* methylation was negatively correlated with *EZH2* expression in AML patients (*R* = −.240, *P* = .002, Figure 1C).

Among a total of 200 AML patients, *EZH1* mutation was identified none of the patients (0%, 0/200), whereas three of the patients harboured *EZH2* mutation (1.5%, 3/200). Clinical/laboratory characteristics of patients with *EZH2* mutation were further presented in Table 1. Interestingly, patients with *EZH2* mutation showed a little lower expression of *EZH2* as compared with patients with *EZH2* wild‐type (*P* = .089, Figure 1D).

**Table 1:** Clinical and laboratory features of AML patients with *EZH2* mutation in AML

Patients ID	EZH2 mutation type (GRCh37)	Sex/Age	FAB	BM blasts	WBC	Cytogenetics	Other mutations
TCGA‐AB‐2817	p.E740Afs*24 p.I739Mfs*25	Male/63y	M2	57%	77.3	45,XY,‐7,t(9;22)(q34;q11.20)[19]/46,XY[1]	BRINP3, TRNT1, ZNF502, GALNT7, CMYA5, GPR6, FAM115A, CYP26A, AP3S2, CBFB, SSTR4
TCGA‐AB‐2865	p.X727_splice	Male/75y	M1	40%	6.4	47,XY,+21[11]/48,XY,+3,+21[8]	PRAMEF2, MYOM3, SLC27A3, MYOC, CLDN18, **TET2**, TTBK1, SLC26A3, PLXNA4, PKHD1L1, JAK2, **KRAS**, DIS3, MYH4, PSG9, **RUNX1**, CACNG2, ATP2B3
TCGA‐AB‐2887	p.R685H	Female/60y	M1	87%	46.5	46,XX,del(7)(q11.2)[20]	**NRAS**, **IDH1**, TMEM18, **DNMT3A**, PRKD3, TMEM108, LAMA2, DYNC2H1, ACAT1, FGD6, BDKRB2, CES1P1, ABCC3, CYP4F2, BRWD3

Bold entries indicate important mutations in AML.

### 3.2 Correlation of EZH2 dysregulation with clinic‐pathologic characteristics in AML

Firstly, we compared the clinical/laboratory features between patients with low and high expression of *EZH2* divided by the median level of *EZH2* expression, and results were shown in Table 2. Patients with lower expression of *EZH2* had significantly older age and higher white blood cells than patients with higher expression of *EZH2* (*P* = .033 and .038). In addition, significant differences in the distributions of French‐American‐British (FAB) classifications and cytogenetics were found between patients with lower and higher expression of *EZH2* (*P* = .016 and .007). Lower expression of *EZH2* was significantly related to FAB‐M5 and −7/del(7) (*P* = .005 and .007). Moreover, lower expression of *EZH2* was also correlated with *NPM1* mutation, *CEBPA* wild‐type and *WT1* wild‐type (*P* = .004, .048 and .009).

**Table 2:** Correlation of *EZH2* dysregulation with clinic‐pathologic characteristics in AML

Patient's parameters	*EZH2* mutation			*EZH2* expression		
	Mutant (n = 3)	Wild‐type (n = 197)	*P*	Low (n = 87)	High (n = 86)	*P*
Sex, male/female	2/1	106/91	1.000	48/39	44/42	.649
Median age, y (range)	63 (60‐75)	57 (18‐88)	.242	60 (18‐81)	54.5 (21‐88)	**.033**
Median WBC, ×10^9^/L (range)	46.5 (6.4‐77.3)	16 (0.4‐298.4)	.422	22.2 (1‐297.4)	13.95 (0.4‐223.8)	**.038**
Median PB blasts, % (range)	65 (48‐70)	33.5 (0‐98)	.218	31.5 (0‐98)	40.5 (0‐97)	.636
Median BM blasts, % (range)	57 (40‐87)	73 (39‐100)	.522	75.5 (30‐99)	71.5 (30‐100)	.218
FAB classifications			.937			**.016**
M0	0	19		10	6	
M1	2	44		20	24	
M2	1	43		15	23	
M3	0	20		4	12	
M4	0	41		20	14	
M5	0	22		**15 (17%)**	**3 (3%)**	**.005**
M6	0	3		1	1	
M7	0	3		1	2	
No data	0	2		1	1	
Cytogenetics			**.028**			**.007**
normal	0	87		40	36	
t(15;17)	0	18		4	11	
t(8;21)	0	7		1	6	
inv(16)	0	12		3	7	
+8	0	10		3	5	
del(5)	0	1		1	0	
−7/del(7)	**2 (67%)**	**7 (4%)**	**.005**	**8 (9%)**	**0 (0%)**	**.007**
11q23	0	4		1	2	
others	1	21		9	10	
complex	0	27		15	9	
No data	0	3		2	0	
Gene mutation						
FLT3 (±)	0/3	56/141	.561	24/63	25/61	.867
NPM1 (±)	0/3	54/143	.565	33/54	15/71	**.004**
DNMT3A (±)	1/2	48/149	.572	25/62	17/69	.215
IDH2 (±)	0/3	20/177	1.000	10/77	7/79	.611
IDH1 (±)	1/2	18/179	.260	9/78	7/79	.794
TET2 (±)	1/2	16/181	.235	9/78	6/80	.590
RUNX1 (±)	1/2	16/181	.235	5/82	10/76	.188
TP53 (±)	0/3	16/181	1.000	8/79	6/80	.782
NRAS (±)	1/2	14/183	.210	8/79	4/82	.370
CEBPA	0/3	13/184	1.000	3/84	10/76	**.048**
WT1	0/3	12/185	1.000	1/86	9/77	**.009**
PTPN11	0/3	9/188	1.000	4/83	4/82	1.000
KIT	0/3	8/189	1.000	3/84	4/82	.720
U2AF1	0/3	8/189	1.000	4/83	3/83	1.000
KRAS	1/2	7/190	.116	5/82	2/84	.443
SMC1A	0/3	7/190	1.000	2/85	5/81	.278
SMC3	0/3	7/190	1.000	1/86	6/80	.064
PHF6	0/3	6/191	1.000	1/86	4/82	.211
STAG2	0/3	6/191	1.000	2/85	3/83	.682
RAD21	0/3	4/193	1.000	3/84	1/85	.621

Bold entries indicate attached statistical significance.

Abbreviations: AML, acute myeloid leukaemia; BM, bone marrow; FAB, French‐American‐British; PB, peripheral blood; WBC, white blood cells.

Secondly, we compared the clinical/laboratory features between patients with and without *EZH2* mutation. We did not found the association of *EZH2* mutation with clinic‐pathologic characteristics besides cytogenetics (Table 2). *EZH2* mutation was significantly associated with −7/del(7) karyotypes (*P* = .005).

### 3.3 Prognostic value of EZH2 dysregulation in AML

**Figure 2:**
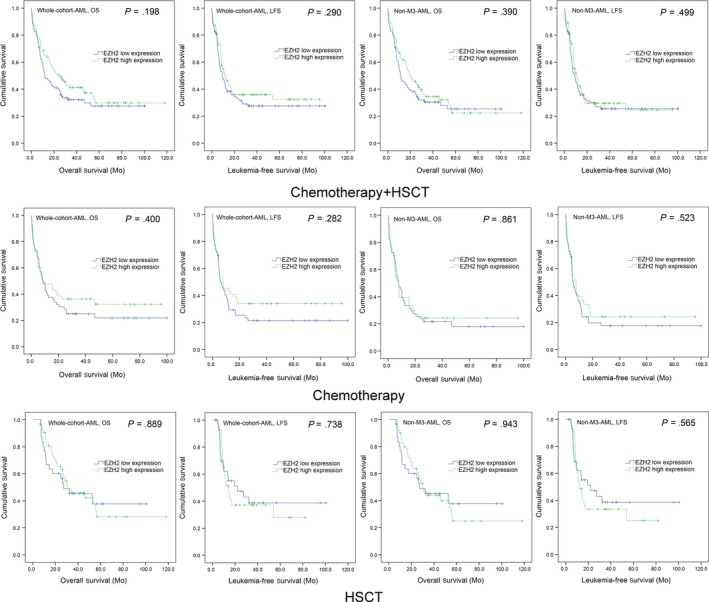
The impact of *EZH2* expression on survival of AML patients. Kaplan‐Meier survival curves of OS and LFS analysed in both chemotherapy and HSCT groups. Survival was analysed though Kaplan‐Meier analysis using Log‐rank test

We first analysed the association of *EZH2* expression with prognosis of AML patients. In both whole‐cohort AML and non‐M3‐AML, *EZH2* lower‐expressed patients showed similar OS and LFS time compared with *EZH2* higher‐expressed patients (Figure 2). Moreover, in chemotherapy and HSCT subgroups, *EZH2* lower‐ and higher‐expressed patients also had no significant difference in OS and LFS time (Figure 2).

**Figure 3:**
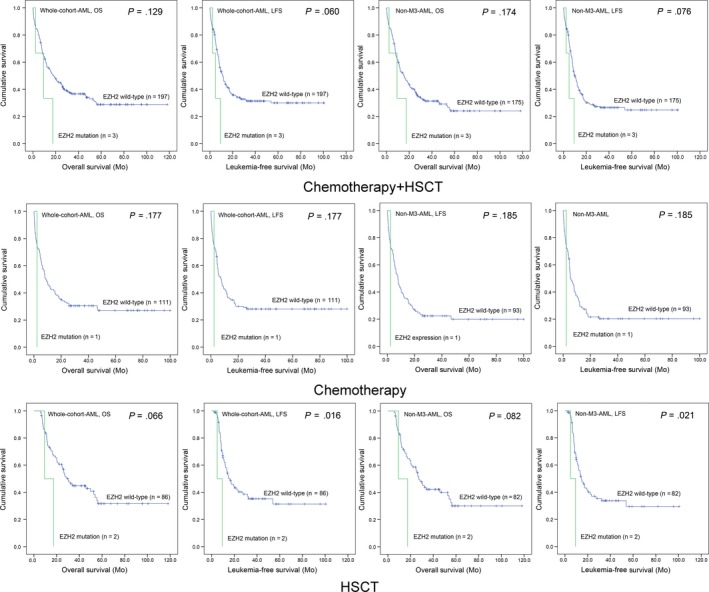
The impact of *EZH2* mutation on survival of AML patients. Kaplan‐Meier survival curves of OS and LFS analysed in both chemotherapy and HSCT groups. Survival was analysed though Kaplan‐Meier analysis using Log‐rank test

Next, we analysed the prognostic effect of *EZH2* mutation on prognosis. In both whole‐cohort AML and non‐M3‐AML patients, although *EZH2* mutant patients showed shorter OS and LFS time compared with *EZH2* wild‐type patients, it did not attach statistic significant (Figure 3). In chemotherapy subgroups, *EZH2* mutant and wild‐type patients also had no significant difference in OS and LFS time among both whole‐cohort AML and non‐M3‐AML patients (Figure 3). However, in HSCT subgroups, significant differences were observed in LFS time and a trend in OS between *EZH2* mutant and wild‐type patients in both whole‐cohort AML and non‐M3‐AML patients (Figure 3).

### 3.4 Low expression of EZH2 in AML benefited from HSCT treatment

**Figure 4:**
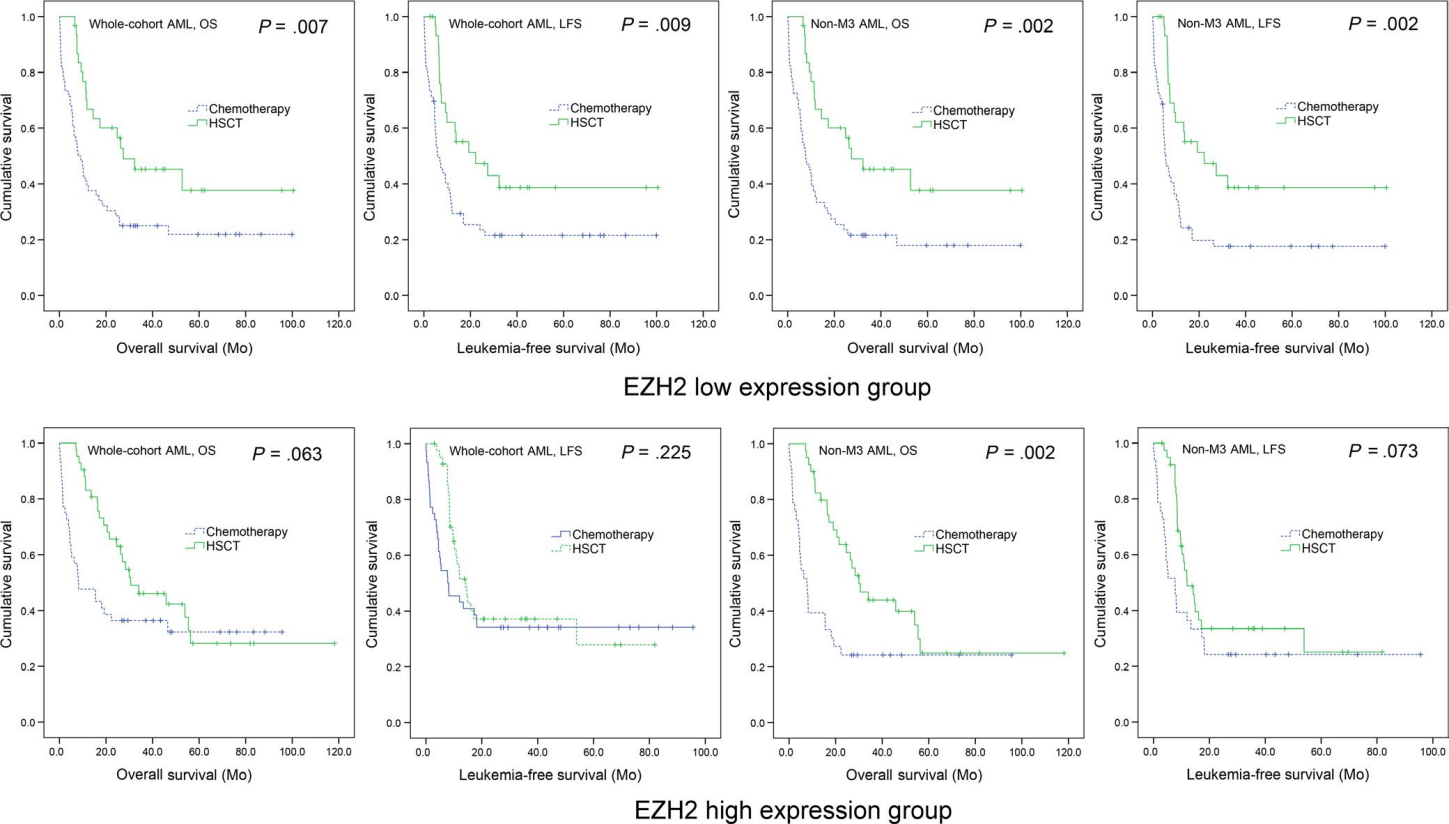
The effect of HSCT on survival of AML patients among *EZH2* low‐ and high‐expressed groups. Kaplan‐Meier survival curves of OS and LFS in low and high *EZH2* expression group. Survival was analysed though Kaplan‐Meier analysis using Log‐rank test

To investigate whether AML patients with abnormal expression of *EZH2* could benefit from HSCT, survival in patients with and without HSCT was compared among both *EZH2* lower‐ and higher‐expressed groups. In the *EZH2* lower‐expressed group, the patients undergoing HSCT had significantly longer OS and LFS compared with patients only received chemotherapy among both whole‐cohort AML and non‐M3‐AML (Figure 4). In the *EZH2* higher‐expressed group, no significant differences in OS and LFS were found between HSCT and chemotherapy groups among both whole‐cohort AML and non‐M3‐AML (Figure 4).

### 3.5 Biologic insights of EZH2 expression in AML

**Figure 5:**
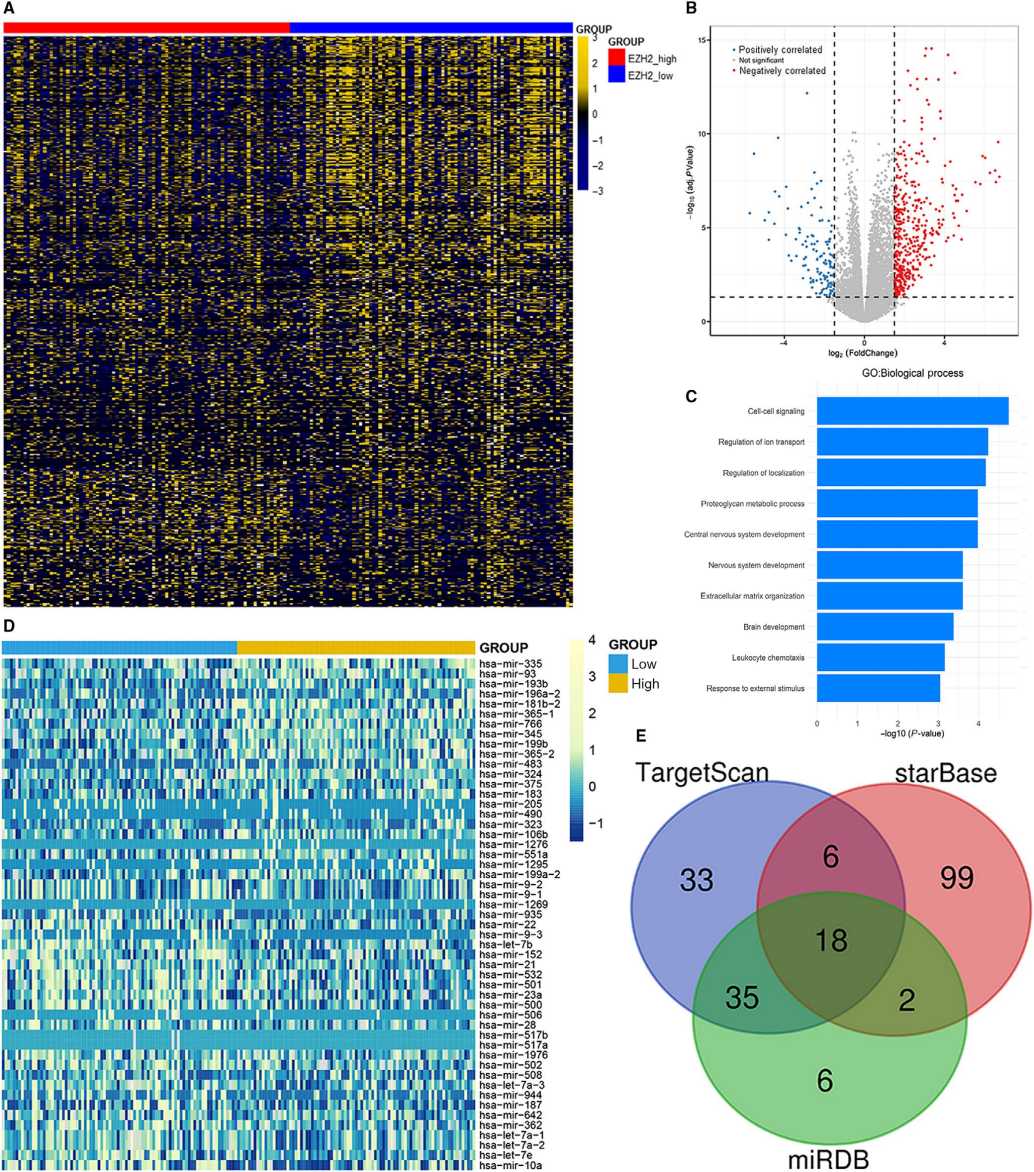
Molecular signatures associated with *EZH2* in AML. A, Expression heat map of differentially expressed genes between *EZH2* low‐ and high‐expressed AML patients (FDR < 0.05, *P* < .05 and |log2 FC|>1.5). B, Volcano plot of differentially expressed genes between *EZH2* low‐ and high‐expressed groups. C, Gene Ontology analysis of differentially expressed genes conducted using online website of STRING (http://string-db.org). D, Expression heat map of differentially expressed microRNAs between *EZH2* low‐ and high‐expressed groups (FDR < 0.05, *P* < .05 and |log2 FC|>0.5). E, Venn results of microRNAs which could target *EZH2* predicted by TargetScan (http://www.targetscan.org/vert_72/), starBase (http://starbase.sysu.edu.cn/agoClipRNA.php?source=mRNA), and miRDB (http://mirdb.org/miRDB/)

In order to identify the molecular network in AML caused by *EZH2* expression abnormalities, we first compared the transcriptomes of *EZH2* lower‐ and higher‐expressed groups. We yielded 568 differentially expressed genes (DEGs), including 136 positively correlated genes and 432 negatively correlated genes (FDR < 0.05, |log2 FC|>1.5; Figure 5A and 5B; Appendix S1). In these DEGs, several cancer‐associated genes such as *HOXC10*, *THBS1*, *CDKN2B*, *PAX2* and *H19* were significantly associated with AML biology. Furthermore, the Gene Ontology analysis revealed that these genes involved in biologic processes, including cell‐cell signalling and leucocyte chemotaxis (Figure 5C).

Next, we also analysed microRNA expression signatures associated with *EZH2* expression. A total of 51 DEGs were identified, including 22 positively correlated genes and 29 negatively correlated genes (FDR < 0.05, |log2 FC|>0.5; Figure 5D; Appendix S2). Negatively correlated microRNAs were *miR‐9*, *miR‐1269*, *miR‐935*, *miR‐22*, *let‐7b*, *miR‐152*, *miR‐21*, *miR‐532*, *miR‐501*, *miR‐23a*, *miR‐500*, *miR‐506*, *miR‐28*, *miR‐517a/b*, *miR‐1976*, *miR‐502*, *miR‐508*, *let‐7a‐3*, *miR‐944*, *miR‐187*, *miR‐642*, *miR‐362*, *let‐7a‐1*, *let‐7a‐2*, *let‐7e* and *miR‐10a*. Of these microRNAs, *miR‐506* was also identified as one of the predicted microRNAs targeting *EZH2* by bioinformatics analysis (Figure 5E, Appendix S3).

## 4 DISCUSSION

Accumulating studies have proved the phenomenon of *EZH2* dysregulation mediated by mutation and expression in diverse human cancers including AML. However, the expression pattern of *EZH2* remains controversial in AML.^22^ Zhu et al showed that overexpression of *EZH2* was a frequent event and was associated with extramedullary infiltration in AML.^17^ In addition, *EZH2* silencing resulted in decreased proliferation and migration ability and increased apoptosis, suggesting its oncogenic role in AML.^17^ In contrast, Göllner et al demonstrated that loss of the histone methyltransferase *EZH2* induced resistance to multiple drugs in AML, indicating it may play as a tumour suppressor gene in AML.^18^ In the current study, we further investigated *EZH2* mutation and expression in AML by the public databases and determined clinical significance. We found *EZH2* mutation was not a frequent event, but *EZH2* under‐expression was a frequent event in AML. Since *EZH2* is located on chromosome 7q36.1, *EZH2* dysregulation was associated with −7/del(7) chromosomal abnormalities.

We next explored the prognostic significance of *EZH2* mutation and expression in AML. Although *EZH2* mutation was not a frequent event in AML, its mutation pattern was associated with poor prognosis in AML patients who received HSCT. In contrast, *EZH2* under‐expression was a frequent event in AML, but its expression was not associated with prognosis in AML. Interestingly, AML patients with *EZH2* under‐expression could significantly benefit from HSCT. These results suggested that *EZH2* expression could serve as a potential biomarker guiding the treatment choice between transplantation and chemotherapy in AML. However, previous study demonstrated that low EZH2 protein levels correlated with poor prognosis in AML patients, which was different from our results.^18^ Moreover, the prognostic effect of *EZH2* mutation in AML was not representative of the results due to the less numbers of *EZH2* mutation in AML. Therefore, further studies are needed to test the prognostic effect of *EZH2* expression on AML, and confirm and expand our results before *EZH2* expression can be used routinely as a potential marker guiding treatment choice in AML patients.

Lastly, we further determined the molecular signatures associated with *EZH2* in AML to further get better understanding of AML biology. We found that *EZH2* dysregulation was significantly associated with *HOX* gene family, *THBS1*, *CDKN2B*, *PAX2* and *H19*, which was reported highly correlated with haematopoiesis and leukaemogenesis.^23^, ^24^, ^25^, ^26^, ^27^ Moreover, for microRNAs, we observed that *EZH2* expression was negatively correlated with several microRNAs such as *miR‐21*, *miR‐23a*, *miR‐500*, *let‐7a‐3*, *miR‐362*, *let‐7e* and *miR‐10a*, which were found to be associated with AML pathogenesis and/or prognosis by previous investigations.^28^, ^29^, ^30^, ^31^, ^32^, ^33^, ^34^, ^35^ Of these microRNAs, *miR‐506* was identified as one of the predicted microRNAs targeting *EZH2* by bioinformatics, which suggested *EZH2* may be a direct target of *miR‐506*. Obviously, further studies are needed to confirm the direct connections of *EZH2* with *miR‐506* by luciferase assay.

Collectively, *EZH2* dysregulation caused by mutation and under‐expression identifies specific subtypes of AML *EZH2* mutation predicts clinical outcome in AML, whereas *EZH2* expression may guide the treatment choice between transplantation and chemotherapy.

## CONFLICT OF INTEREST

The authors declare that they have no competing interests.

## AUTHOR CONTRIBUTIONS

Jing‐dong Zhou and Jun Qian conceived and designed the experiments; Ming‐qiang Chu, Ting‐juan Zhang and Zi‐jun Xu analysed the data; Yu Gu, Ji‐chun Ma, Wei Zhang, Xiang‐mei Wen and Jiang Lin offered technique support; Jing‐dong Zhou wrote the paper. All authors read and approved the final manuscript.

## ETHICAL APPROVAL

The present study approved by the Ethics Committee and Institutional Review Board of the Affiliated People's Hospital of Jiangsu University and the Washington University Human Studies Committee.

## CONSENT TO PARTICIPATE

Written informed consents were obtained from all enrolled individuals prior to their participation.

## Data Availability

The processed and normalized datasets supporting the conclusions of this article are included within the article (File [Link], [Link], [Link]). Raw data used during the current study are available from the corresponding author upon reasonable request.
